# Lung Cancer Detection Using Image Segmentation by means of Various Evolutionary Algorithms

**DOI:** 10.1155/2019/4909846

**Published:** 2019-01-08

**Authors:** K. Senthil Kumar, K. Venkatalakshmi, K. Karthikeyan

**Affiliations:** ^1^Assistant Professor, Department of Electrical and Electronics Engineering, University College of Engineering, Arni, India; ^2^Assistant Professor, Department of Electronics and Communication Engineering, University College of Engineering Tindivanam, Tindivanam, India; ^3^Teaching Fellow, Department of Electronics and Communication Engineering, University College of Engineering, Arni, India

## Abstract

The objective of this paper is to explore an expedient image segmentation algorithm for medical images to curtail the physicians' interpretation of computer tomography (CT) scan images. Modern medical imaging modalities generate large images that are extremely grim to analyze manually. The consequences of segmentation algorithms rely on the exactitude and convergence time. At this moment, there is a compelling necessity to explore and implement new evolutionary algorithms to solve the problems associated with medical image segmentation. Lung cancer is the frequently diagnosed cancer across the world among men. Early detection of lung cancer navigates towards apposite treatment to save human lives. CT is one of the modest medical imaging methods to diagnose the lung cancer. In the present study, the performance of five optimization algorithms, namely, *k*-means clustering, *k*-median clustering, particle swarm optimization, inertia-weighted particle swarm optimization, and guaranteed convergence particle swarm optimization (GCPSO), to extract the tumor from the lung image has been implemented and analyzed. The performance of median, adaptive median, and average filters in the preprocessing stage was compared, and it was proved that the adaptive median filter is most suitable for medical CT images. Furthermore, the image contrast is enhanced by using adaptive histogram equalization. The preprocessed image with improved quality is subject to four algorithms. The practical results are verified for 20 sample images of the lung using MATLAB, and it was observed that the GCPSO has the highest accuracy of 95.89%.

## 1. Introduction

Lung cancer, also known as lung carcinoma, is a malignant tumor characterized by uncontrolled growth of the cell in tissues of the lung. It is mandatory to treat this to avoid spreading its growth by metastasis to other parts of the body. Most cancers that start in the lung are carcinomas. The two main types are small-cell lung carcinoma and non-small-cell lung carcinoma [1]. Long-period tobacco smoking is the primary factor for 85% of lung cancers [2]. About 10–15% of cases occur in people who have never smoked but due to air pollution, secondhand smoking, asbestos, and radon gas. Computer tomography (CT) and radiographs are the conventional methods to detect the presence of lung cancer. The diagnosis is confirmed by biopsy which is usually performed by bronchoscopy or CT scan. The cause of cancer-related death among men is mainly due to lung cancer. Hence, it is essential to determine a new robust method to diagnose the lung cancer at an earlier stage [3]. For the present study, 20 lung image samples and four algorithms have been taken for analysis. It was proved that the combination of adaptive median filter, adaptive histogram equalization, and guaranteed convergence particle swarm optimization- (GCPSO-) based algorithm has more accurate results among others.

## 2. Methods

**Figure 1:**
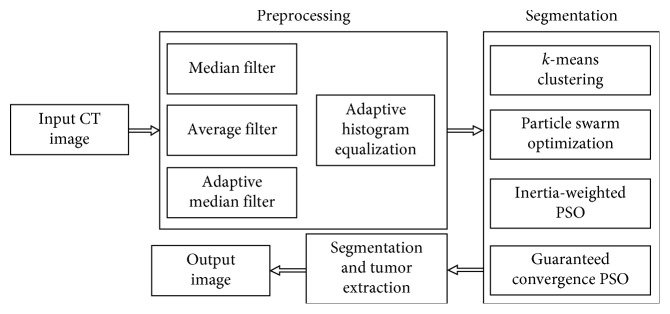
Process flow diagram of the projected method.

In medical image segmentation, the accuracy is foremost important, as it deals with human lives. It is highly crucial to eradicate the incidence of noise content and to improve the image quality before an examination [4]. This part of work is known as preprocessing. In the preprocessing stage, noise removal and contrast enhancement are two primary steps. In the present study, the performance results of median, adaptive median, and average filters to isolate the presence of speckle noise have been compared. The coding for the same has been implemented using MATLAB. Furthermore, the image quality and visual appearance are improved by adaptive histogram equalization. The second stage of work is segmentation. This stage consists of applying five methods, namely, *k*-means, *k*-median, particle swarm optimization (PSO), inertia-weighted particle swarm optimization (IWPSO), and GCPSO. The tumor portion was extracted from the segmented results of the above-said five methods and compared with manual extraction. The results show that the GCPSO-based segmentation has more accuracy than the others.  Figure 1 depicts the process of operation for the present study.

### 2.1. Median and Adaptive Median Filters

The median filter removes the noise and retains the sharpness of the image. Accordance to the name, each pixel is replaced by the median value from the neighborhood pixels. A 3 × 3 window is used in this filter [5]. This is one of the best filters among conventional filters which remove the speckle noise. The steps followed to construct the median filter are given in Algorithm 1.

Spatial processing to preserve the edge detail and to eliminate nonimpulsive noise by the adaptive median filter plays a vital role. The small structure in the image and edges are retained by the adaptive median filter. In the adaptive median filter, the window size varies with respect to each pixel.

### 2.2. Average Filter

This is a simple filter which removes the spatial noise from a digital image. The presence of spatial noise is mainly due to the data acquisition process. The neighborhood mean value is measured for each and every pixel and is replaced by the corresponding mean value. This process is repeated for every pixel in the image [5]. All the pixels in the digital image are modified by sliding the operator over the entire range of pixels. The steps followed for the average filter are given in Algorithm 2.

### 2.3. Histogram Equalization

Image enhancement is the technique which is used to improve the image quality. For better understanding and analysis, it is mandatory to enhance the contrast of medical images. The conventional method used for this operation is histogram equalization. A minor adjustment on the intensity of image pixels is done in this method. Each pixel is mapped to intensity proportional to its rank in the surrounding pixels. The steps followed for histogram equalization are given in Algorithm 3 [6].

### 2.4. *k*-Means Clustering Algorithm

The simplest and conventional method in cluster analysis is the *k*-means clustering algorithm. This algorithm segregates the given dataset into two or more clusters [7]. The accuracy of this method completely depends on the selection of the cluster center. It is mandatory to select the optimum cluster center to get a better result. The Euclidean distance is the general measure to segregate the dataset [8]. Pixels are assigned to an individual cluster based on the Euclidean distance. The objective function used in this algorithm is

(1) $$\begin{array}{c} J\left(v\right)=\overset{C}{\underset{i=1}{\sum }}\overset{Ci}{\underset{j=1}{\sum }}{\left(\left\|x_{i}-v_{j}\right\|\right)}^{2}, \end{array}$$

where *x*_*i*_ are the pixels, *v*_*j*_ are the cluster centers, ‖*x*_*i*_ − *v*_*j*_‖ is the Euclidean distance between *x*_*i*_ and *v*_*j*_, *C*_*i*_ is the number of data points for the *i*th cluster, and *C* is the number of cluster centers [9]. The steps followed for k-means clustering are given in Algorithm 4.

### 2.5. *k*-Median Clustering Algorithm

This is also a clustering algorithm slightly modified from the *k*-means algorithm. In centroid calculation instead of calculating the mean value, the median value is considered. This algorithm significantly reduces the error since there is no squared operation as in the calculation of the Euclidean distance. The clusters formed by this method are more compact. As an alternate, this approach uses the Lloyd-style iteration. The steps followed for *k*-median clustering are given in Algorithm 5 [10].

### 2.6. Particle Swarm Optimization

PSO is a metaheuristic algorithm used efficiently in medical image analysis [11]. It mimics the social behavior of the birds searching for food [12]. The fundamental idea of PSO is sharing and communicating the information. In this approach, each particle has initial position and velocity. Based on the fitness value, the velocity and position are updated. The relevant two equations in PSO to update the position and velocity are as follows [11, 12]:

(2) $$\begin{array}{c} v\left(t+1\right)=v\left(t\right)+c_{1}r_{1}\left[\text{pbest}\left(t\right)-x\left(t\right)\right]+c_{2}r_{2}\left[\text{gbest}\left(t\right)-x\left(t\right)\right],\\ x\left(t+1\right)=x\left(t\right)+v\left(t+1\right), \end{array}$$

where *r*_1_ and *r*_2_ are the random numbers and the acceleration coefficients *c*_1_ and *c*_2_ are two positive constants. The success of PSO relies on the fitness function. The following fitness function has been used for the present study:

(3) $$\begin{array}{c} \text{maximize }f=\overset{n}{\underset{i=1}{\sum }}\frac{\text{intercluster distance}}{\text{intracluster distance}}, \end{array}$$

where *n* is the number of clusters. The steps followed for the particle swarm optimization are shown in Algorithm 6.

### 2.7. Inertia-Weighted Particle Swarm Optimization

The exploration and exploitation in PSO are based on the inertia weight. The basic PSO, presented by Eberhart and Kennedy in 1995, has no inertia weight. In 1998, Shi and Eberhart introduced the concept of inertia weight by adding constant inertia weight. They stated that a significant inertia weight facilitates a global search, while a small inertia weight facilitates a local search [14]. This enhances the convergence rate and reduces the number of iterations. Inertia weight less than 1, in general, improves the results. The used method improves the convergence rate and saves the time taken and some iterations.

The resulting velocity update equation becomes

(4) $$\begin{array}{c} v\left(t+1\right)=w\;*\;v\left(t\right)+c_{1}r_{1}\left[\text{pbest}\left(t\right)-x\left(t\right)\right]+c_{2}r_{2}\left[\text{gbest}\left(t\right)-x\left(t\right)\right], \end{array}$$

where *w* is the inertia weight, with constant inertia weight *w* = 0.7 and random inertia weight *w* = 0.5 + rand()/2.

### 2.8. Guaranteed Convergence Particle Swarm Optimization

The GCPSO focuses on a new particle which deals with the current best position in the region. In this task, this particle is treated as a member of the swarm, and the velocity update equation for this new particle is given as follows [15]:

(5) $$\begin{array}{c} v\varphi \left(t+1\right)=x\varphi \left(t\right)+\text{pbest}\left(t\right)+\omega v\varphi \left(t\right)+\rho \left(t\right)\left(1-2r\right). \end{array}$$

The search ability is increased by the social part. This will improve the random search in the area around the gbest position. The random vector and diameter of the search area are *r* and *ρ*(*t*), respectively. The range of the random vector lies between 0 and 1. The diameter of the search area can be updated using the following equation:

(6) $$\begin{array}{c} \rho \left(t+1\right)=\left\{\begin{array}{cc} 2\rho \left(t\right), & \#\text{successes}>\text{sc,}\\ \left(\frac{1}{1.5}\right)\rho \left(t\right), & \#\text{failures}>\text{fc,}\\ \rho \left(t\right), & \text{otherwise,} \end{array}\right. \end{array}$$

where the terms #successes and #failures are defined as the number of consecutive successes and failures, respectively. The threshold parameters sc and fc are determined empirically. Since it is hard to obtain a better value in only a few iterations in a high-dimensional search space, the recommended values are thus sc = 15 and fc = 5. On some benchmark tests, the GCPSO has shown an excellent performance of locating the minimal of a space after unimodal with only a small amount of particles. The steps to be followed for the GCPSO are shown in Algorithm 7.

## 3. Performance Measures

**Figure 2:**
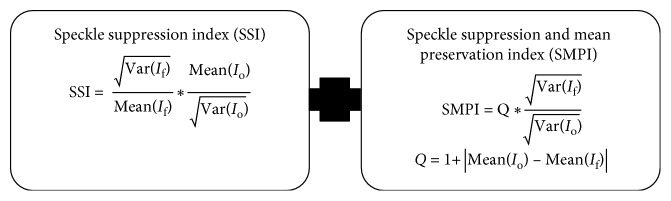
Performance measures of the filter.

Certain performance measures are used to evaluate the results obtained from medical image segmentation. The list of performance measures used to assess the filter operation is shown in Figure 2 [16]. Let *I*_f_ be the image after noise reduction and *I*_0_ be the noisy image.

**Figure 3:**
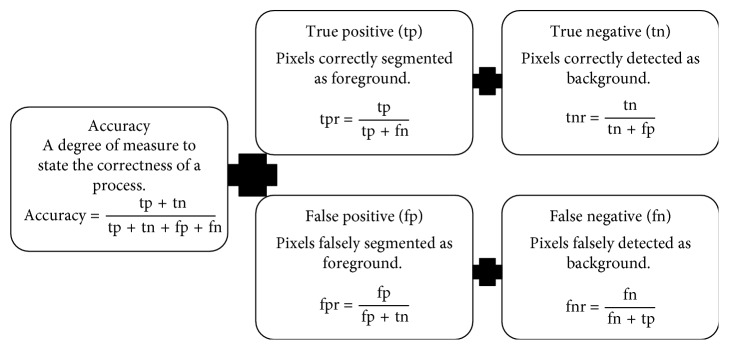
Performance measures for the medical image segmentation.

Performance measures used for the evaluation of the results of the segmentation algorithm are given in Figure 3 [17].

## 4. Results and Discussion

The used methods are practically implemented using MATLAB coding, and the results were verified.

**Figure 4:**
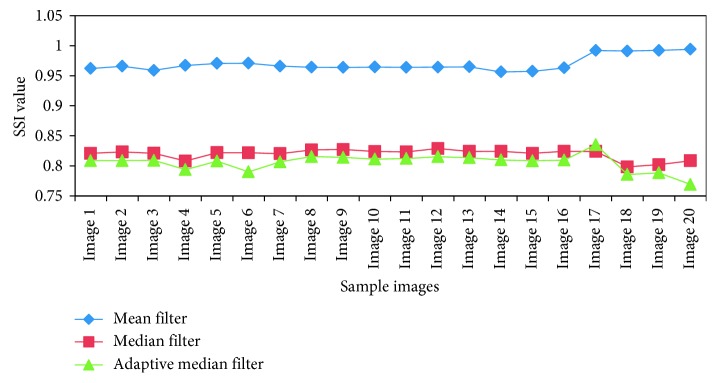
Comparative results of SSI values.

**Figure 5:**
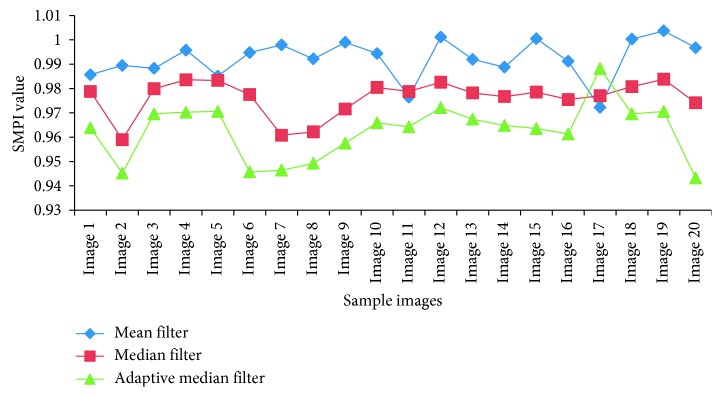
Comparative results of SMPI values.

In the preprocessing stage, a comparison was done between the performance of median, adaptive median, and mean filters. The SSI and SMPI values are shown in Table 1 and Figures 4 and 5. From the results, it is evident that the adaptive median filter has accurate characteristics than the mean and median filters for medical image segmentation.

The segmentation accuracy was measured using the true positive rate, true negative rate, false positive rate, and false negative rate by comparing the results from the algorithm with manual segmentation results. The practical results of the *k*-means clustering segmentation algorithm are shown in Table 2.

The practical results of the *k*-median clustering segmentation algorithm are shown in Table 3.

The practical results of the PSO-based segmentation algorithm are shown in Table 4.

The practical results of the IWPSO segmentation algorithm are shown in Table 5.

The practical results of the GCPSO segmentation algorithm are shown in Table 6.

**Figure 6:**
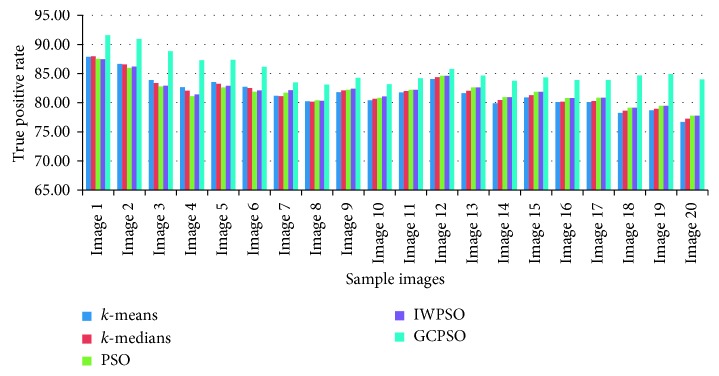
Comparative results of the true positive rate value.

**Figure 7:**
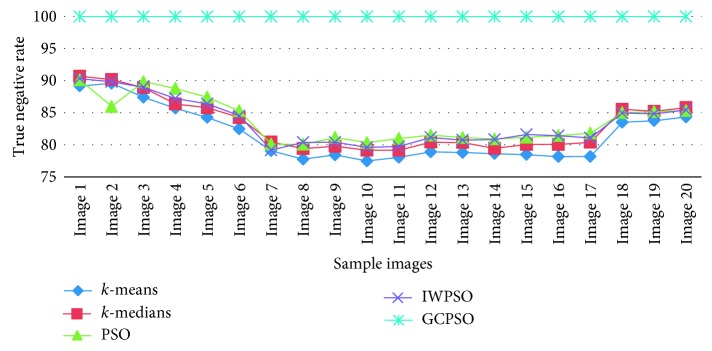
Comparative results of the true negative rate value.

**Figure 8:**
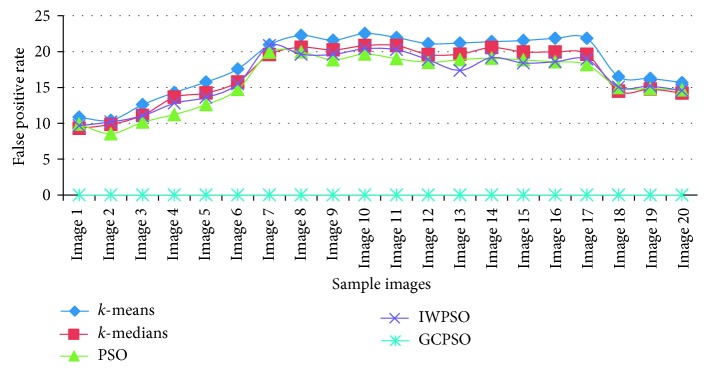
Comparative results of the false positive rate value.

**Figure 9:**
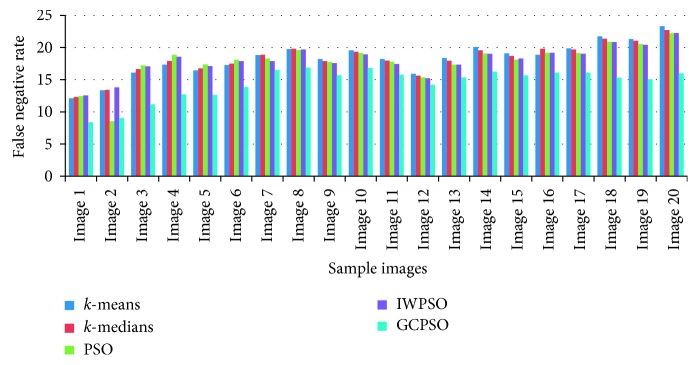
Comparative results of the false negative rate value.

The graphical view of the comparison of the true positive rate, true negative rate, false positive rate, and false negative rate for the algorithms used is shown in Figures 6–9. It is proved that the true positive and true negative rates are high and false positive and false negative rates are low for the GCPSO algorithm.

**Figure 10:**
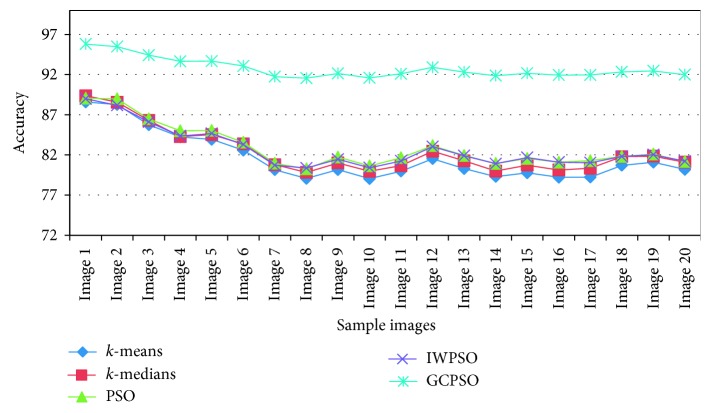
Comparative results of accuracy.

The comparative evaluation based on the accuracy of the segmentation is shown in Table 7 and Figure 10. The results indicate that the GCPSO-based technique has the highest average value of accuracy than the other methods.

**Figure 11:**
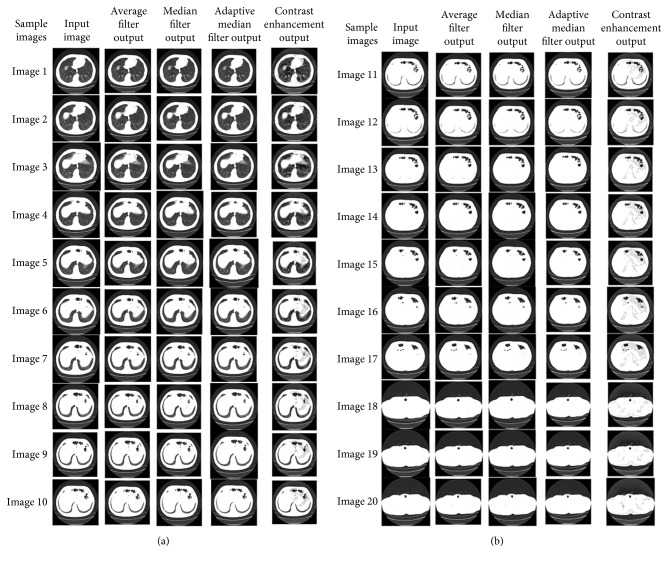
Resultant images after preprocessing.

The resultant images after preprocessing are shown in Figures 11(a) and 11(b).

**Figure 12:**
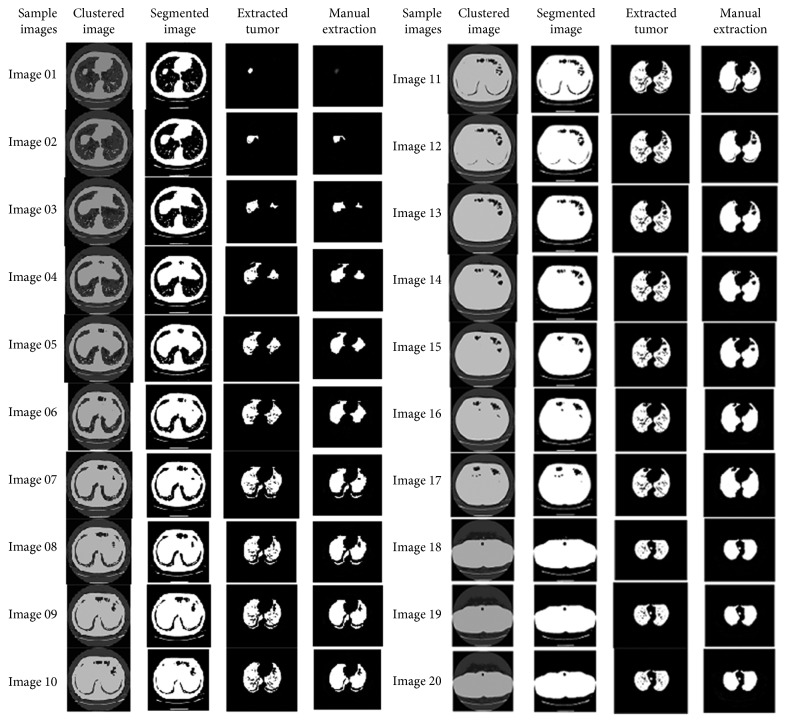
Resultant images by *k*-means clustering.

The resultant images after segmentation using *k*-means clustering are shown in Figure 12.

**Figure 13:**
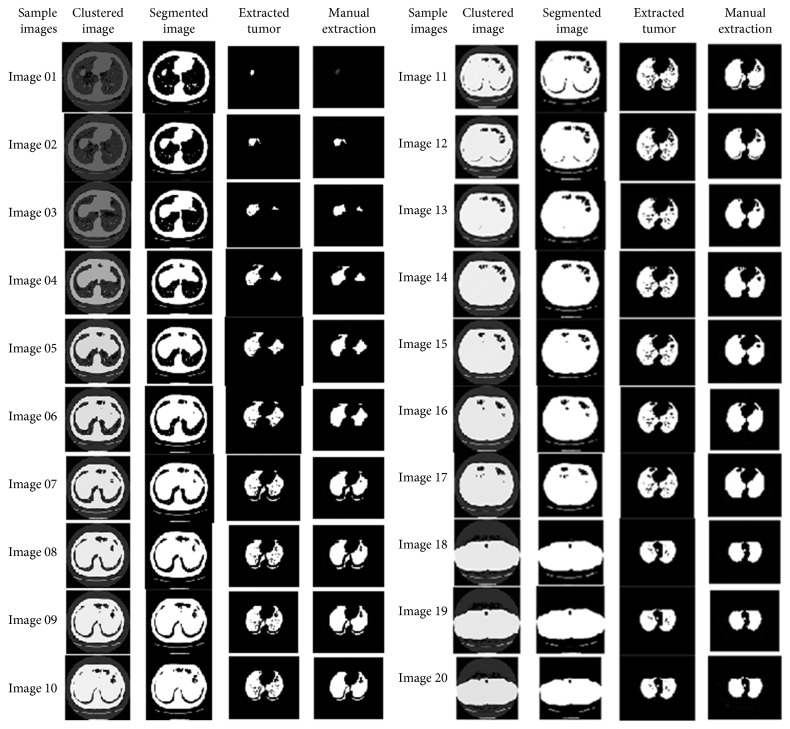
Resultant images by *k*-median clustering.

The resultant images after segmentation using *k*-median clustering are shown in Figure 13.

**Figure 14:**
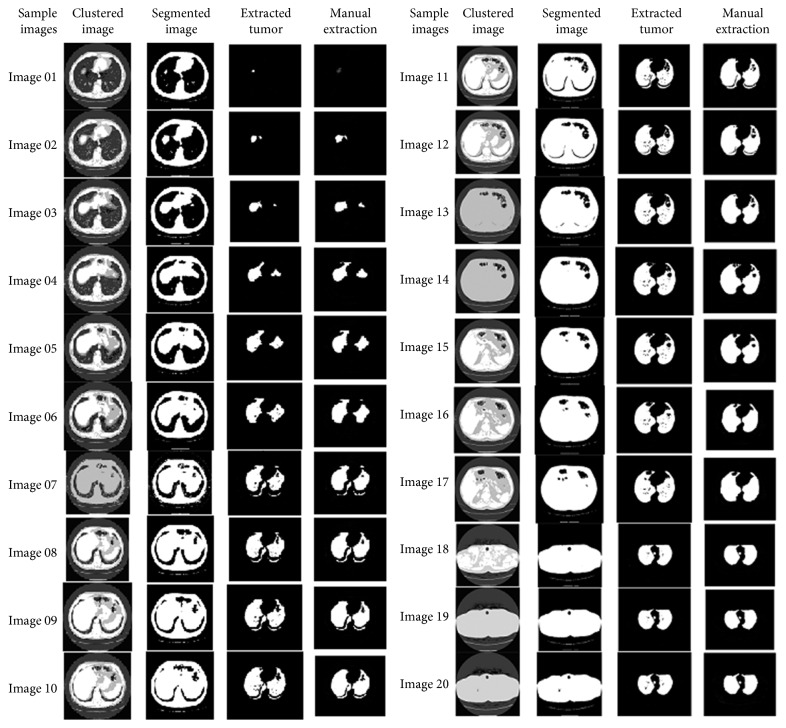
Resultant images by the PSO algorithm.

The resultant images after segmentation using the PSO algorithm are shown in Figure 14.

**Figure 15:**
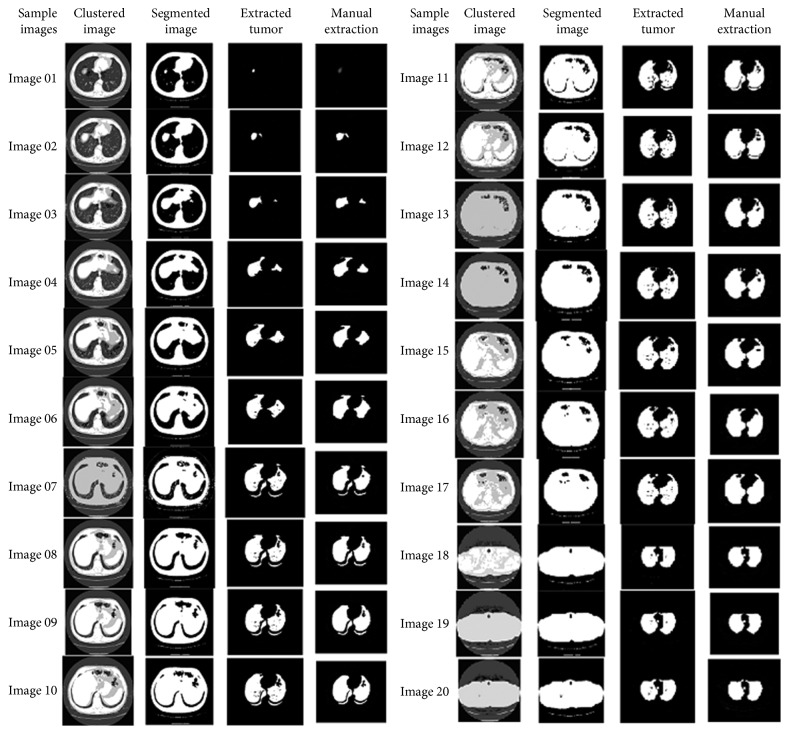
Resultant images by IWPSO algorithm clustering.

The resultant images after segmentation using the IWPSO algorithm are shown in Figure 15.

**Figure 16:**
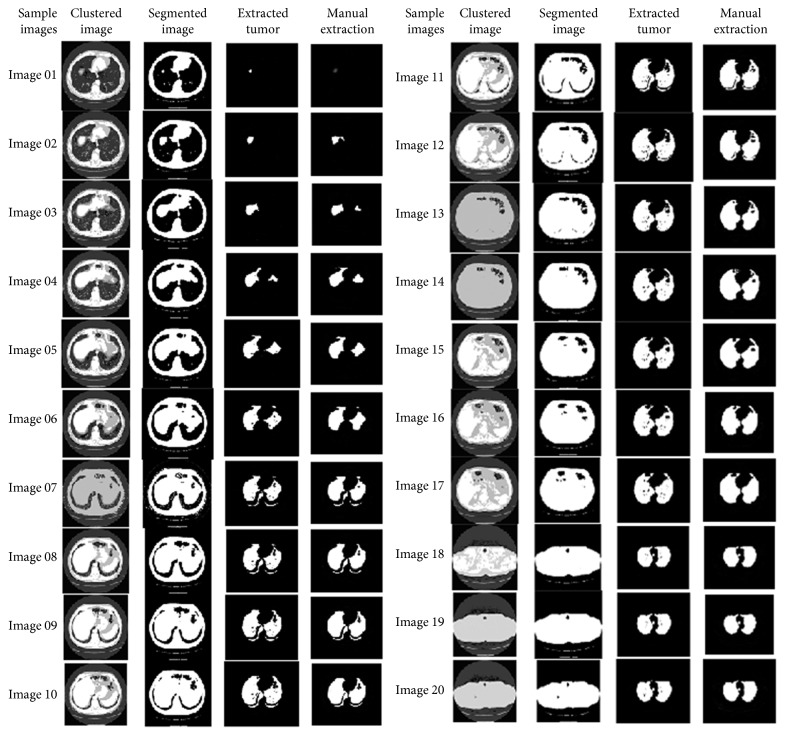
Resultant images by GCPSO algorithm clustering.

The resultant images after segmentation using the GCPSO algorithm are shown in Figure 16.

In an earlier research, lung cancer detection was done using PSO, genetic optimization, and SVM algorithm with the Gabor filter and produced an accuracy of 89.5% [18]. The method to detect lung cancer by means of K-NN classification using the genetic algorithm produced a maximum accuracy of 90% [19]. The comparative results with respect to the above-said methods are shown in Table 8.

**Figure 17:**
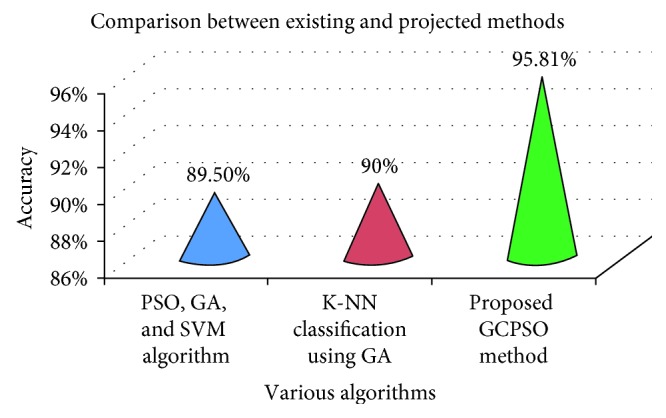
Graphical view of accuracy.

The graphical comparative analysis between the used and existing methods is shown in Figure 17.

## 5. Conclusion

In this study, various optimization algorithms have been evaluated to detect the tumor. Medical images often need preprocessing before being subjected to statistical analysis. The adaptive median filter has better results than median and mean filters because the speckle suppression index and speckle and mean preservation index values are lower for the adaptive median filter. Comparing the five algorithms, the accuracy of the tumor extraction is improved in GCPSO with the highest accuracy of 95.8079%, and it obtained above 90% of precision in all the 20 images. It is more accurate when compared to the previous method which had an accuracy of 90% in 4 out of 10 datasets only. In future studies, the use of more number of optimization algorithms will be included to improve the accuracy.

## Figures and Tables

**Algorithm 1:**
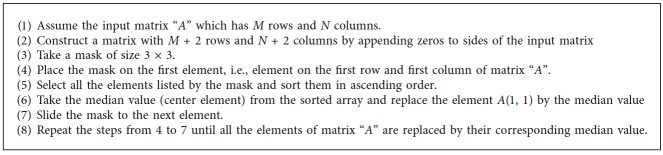
Median filter.

**Algorithm 2:**
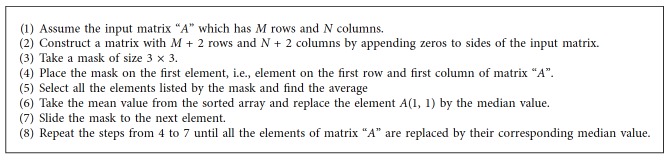
Median filter.

**Algorithm 3:**
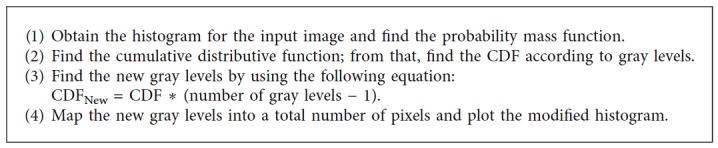
Histogram equalization.

**Algorithm 4:**
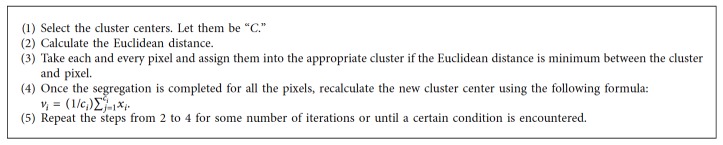
*k*-Means clustering.

**Algorithm 5:**
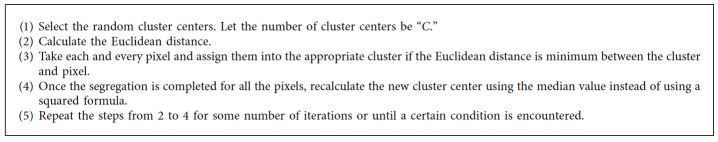
*k*-Median clustering.

**Algorithm 6:**
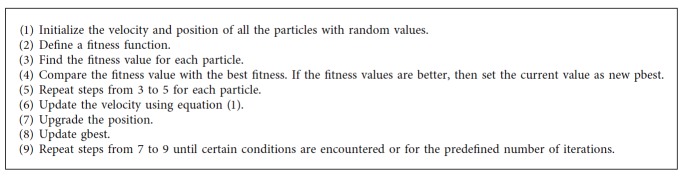
Particle swarm optimization [11, 13].

**Algorithm 7:**
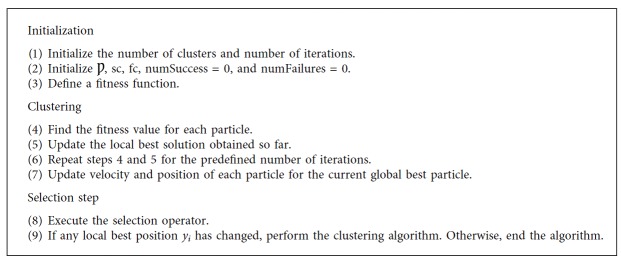
GCPSO algorithm [15].

**Table 1:** SSI and SMPI values of input images.

Sample images	SSI			SMPI		
	Mean filter	Median filter	Adaptive median filter	Mean filter	Median filter	Adaptive median filter
Image 1	0.9621	0.8208	0.8086	0.9857	0.9788	0.9638
Image 2	0.9658	0.8232	0.8087	0.9895	0.959	0.9452
Image 3	0.9588	0.8209	0.8091	0.9883	0.9799	0.9696
Image 4	0.9671	0.8080	0.7937	0.9958	0.9836	0.9703
Image 5	0.9705	0.8220	0.8078	0.9851	0.9833	0.9706
Image 6	0.9708	0.8218	0.7900	0.9948	0.9775	0.9457
Image 7	0.9660	0.8202	0.8067	0.9979	0.9608	0.9464
Image 8	0.9640	0.8265	0.8154	0.9922	0.9622	0.9493
Image 9	0.9638	0.8272	0.8141	0.9990	0.9716	0.9576
Image 10	0.9644	0.8238	0.8112	0.9944	0.9804	0.9659
Image 11	0.9639	0.8231	0.8122	0.9765	0.9788	0.9643
Image 12	0.9642	0.8289	0.8152	1.0012	0.9826	0.9721
Image 13	0.9648	0.8239	0.8135	0.9920	0.9782	0.9674
Image 14	0.9564	0.8242	0.8098	0.9888	0.9767	0.9648
Image 15	0.9573	0.8208	0.8084	1.0005	0.9785	0.9636
Image 16	0.9631	0.8242	0.8095	0.9912	0.9755	0.9613
Image 17	0.9919	0.8239	0.8352	0.9722	0.9770	0.9882
Image 18	0.9912	0.7983	0.7857	1.0003	0.9808	0.9696
Image 19	0.9921	0.8020	0.7884	1.0037	0.9838	0.9706
Image 20	0.9939	0.8085	0.7690	0.9968	0.9741	0.9432

**Table 2:** Statistical results from the *k*-means algorithm.

Images	True positive rate	True negative rate	False positive rate	False negative rate	Accuracy
Image 1	87.8783	89.1554	10.8446	12.1217	88.5937
Image 2	86.6527	89.5874	10.4126	13.3473	88.2682
Image 3	83.8975	87.3900	12.6100	16.1025	85.7501
Image 4	82.6502	85.7011	14.2989	17.3498	84.2186
Image 5	83.5680	84.2582	15.7418	16.4320	83.9216
Image 6	82.7250	82.4643	17.5654	17.2750	82.5795
Image 7	81.1893	79.0554	20.9446	18.8107	80.1519
Image 8	80.2543	77.7549	22.2451	19.7457	79.0656
Image 9	81.7874	78.4139	21.5861	18.2126	80.1606
Image 10	80.4304	77.4794	22.5206	19.5696	79.0378
Image 11	81.7725	78.0352	21.9648	18.2275	79.9755
Image 12	84.0795	78.8912	21.1088	15.9205	81.5238
Image 13	81.6145	78.7989	21.2011	18.3855	80.2806
Image 14	79.8951	78.6152	21.3848	20.1049	79.3023
Image 15	80.9012	78.4626	21.5374	19.0988	79.7600
Image 16	80.1249	78.1480	21.8520	18.8751	79.2121
Image 17	80.1220	78.1687	21.8318	19.8780	79.2229
Image 18	78.2509	83.5148	16.4852	21.7491	80.7020
Image 19	78.7041	83.7431	16.2569	21.2959	81.0816
Image 20	76.7118	84.3245	15.6755	23.2882	80.1831

**Table 3:** Statistical results from the *k*-median clustering segmentation algorithm.

Images	True positive rate	True negative rate	False positive rate	False negative rate	Accuracy
Image 1	87.9631	90.6864	9.3136	12.3069	89.3672
Image 2	86.5908	90.1719	9.8281	13.4092	88.5622
Image 3	83.3821	88.9051	11.0949	16.6179	86.2969
Image 4	82.0844	86.3637	13.6363	17.9156	84.2695
Image 5	83.2410	85.7769	14.2231	16.7590	84.5294
Image 6	82.5053	84.2412	15.7588	17.4947	83.3654
Image 7	81.1107	80.4281	19.5719	18.8893	80.7832
Image 8	80.1857	79.4033	20.5967	19.8143	79.8186
Image 9	82.1213	79.7647	20.2353	17.8787	80.9977
Image 10	80.6627	79.1577	20.8423	19.3373	79.9611
Image 11	82.0209	79.1588	20.8412	17.9791	80.6621
Image 12	84.3809	80.4121	19.5879	15.6191	82.4514
Image 13	82.0487	80.3496	19.6504	17.9513	81.2545
Image 14	80.4506	79.4375	20.5625	19.5494	79.9876
Image 15	81.3002	80.0536	19.9464	18.6998	80.7248
Image 16	80.1942	80.0503	19.9497	19.8058	80.1291
Image 17	80.2984	80.3756	19.6244	19.7016	80.3332
Image 18	78.6327	85.5226	14.4774	21.3673	81.7792
Image 19	78.9322	85.2163	14.7837	21.0678	81.8439
Image 20	77.2752	85.8000	14.2000	22.7248	81.0973

**Table 4:** Statistical results from the PSO algorithm.

Images	True positive rate	True negative rate	False positive rate	False negative rate	Accuracy
Image 1	87.5413	90.1196	9.8804	12.4587	89.0624
Image 2	85.9612	85.9612	8.5479	8.5479	88.9689
Image 3	82.7919	89.8314	10.1686	17.2081	86.4850
Image 4	81.1271	88.7838	11.2162	18.8729	84.9967
Image 5	82.6343	87.3995	12.6005	17.3657	85.0299
Image 6	81.8996	85.2900	14.7100	18.1004	83.5581
Image 7	81.7281	80.0949	19.9051	18.2719	80.9438
Image 8	80.4182	80.0721	19.9279	19.5818	80.2571
Image 9	82.2573	81.1450	18.8550	17.7427	81.7340
Image 10	80.8521	80.3433	19.6567	19.1479	80.6182
Image 11	82.2198	80.9837	19.0163	17.7802	81.6421
Image 12	84.6322	81.5070	18.4930	15.3678	83.1347
Image 13	82.6283	81.1153	18.8847	17.3617	81.9351
Image 14	80.9338	80.9090	19.0910	19.0662	80.9226
Image 15	81.8790	81.1729	18.8271	18.1210	81.5586
Image 16	80.8120	81.4222	18.5778	19.1880	81.0836
Image 17	80.8582	81.8136	18.1864	19.1418	81.2824
Image 18	79.1387	85.0084	14.9916	20.8613	81.8114
Image 19	79.4655	85.1570	14.8430	20.5345	82.0954
Image 20	77.7826	85.3446	14.6554	22.2174	81.1744

**Table 5:** Statistical results from the IWPSO algorithm.

Images	True positive rate	True negative rate	False positive rate	False negative rate	Accuracy
Image 1	87.4649	90.3272	9.6728	12.5351	88.9813
Image 2	86.1950	89.8126	10.1874	13.8050	88.1810
Image 3	82.9347	88.9622	11.0378	17.0653	86.1018
Image 4	81.4285	87.2013	12.7987	18.5715	84.3584
Image 5	82.9023	86.3940	13.6060	17.0977	84.6631
Image 6	82.1065	84.5145	15.4855	17.8935	83.2876
Image 7	82.1361	79.1744	20.8256	17.8639	80.7064
Image 8	80.3274	80.3855	19.6145	19.6726	80.3544
Image 9	82.4185	80.3924	19.6076	17.5815	81.4631
Image 10	81.0769	79.5965	20.4035	18.9231	80.3943
Image 11	82.2198	79.7401	20.2599	17.4299	81.2411
Image 12	84.6322	81.1390	18.8610	15.2172	83.0334
Image 13	82.6283	80.7231	17.3596	17.3596	81.8905
Image 14	80.9338	80.8231	19.1769	19.0328	80.9025
Image 15	81.8790	81.5899	18.4101	18.2694	81.6669
Image 16	80.8120	81.4173	18.5827	19.1734	81.0896
Image 17	80.8582	81.0677	18.9323	19.0166	81.0209
Image 18	79.1387	84.9622	15.0378	20.8310	81.8081
Image 19	79.4655	84.8219	15.1781	20.3936	82.0213
Image 20	77.7684	85.4281	14.5719	22.2316	81.2033

**Table 6:** Statistical results from the GCPSO algorithm.

Images	True positive rate	True negative rate	False positive rate	False negative rate	Accuracy
Image 1	91.6158	99.9999	0.0001	8.3842	95.8079
Image 2	90.9563	99.9999	0.0001	9.0437	95.4782
Image 3	88.8404	99.9999	0.0001	11.1592	94.4204
Image 4	87.2946	99.9999	0.0001	12.7054	93.6473
Image 5	87.3583	99.9999	0.0001	12.6417	93.6792
Image 6	86.1567	99.9999	0.0001	13.8433	93.0784
Image 7	83.4867	99.9999	0.0001	16.5133	91.7434
Image 8	83.1082	99.9999	0.0001	16.8918	91.5541
Image 9	84.2907	99.9999	0.0001	15.7093	92.1453
Image 10	83.1917	99.9999	0.0001	16.8083	91.5958
Image 11	84.2122	99.9999	0.0001	15.7878	92.1061
Image 12	85.7977	99.9999	0.0001	14.2023	92.8988
Image 13	84.6397	99.9999	0.0001	15.3603	92.3198
Image 14	83.7442	99.9999	0.0001	16.2558	91.8721
Image 15	84.3299	99.9999	0.0001	15.6701	92.1649
Image 16	83.8867	99.9999	0.0001	16.1133	91.9433
Image 17	83.9061	99.9999	0.0001	16.0939	91.9531
Image 18	84.6836	99.9999	0.0001	15.3164	92.3418
Image 19	84.9324	99.9999	0.0001	15.0676	92.4662
Image 20	83.9867	99.9999	0.0001	16.0124	91.9938

**Table 7:** Statistical comparative result of accuracy.

Images	*k*-Means	*k*-Median	PSO	IWPSO	GCPSO
Image 1	88.5937	89.3672	89.0624	88.9813	95.8079
Image 2	88.2682	88.5622	88.9689	88.1810	95.4782
Image 3	85.7501	86.2969	86.4850	86.1018	94.4204
Image 4	84.2186	84.2695	84.9967	84.3584	93.6473
Image 5	83.9216	84.5294	85.0299	84.6631	93.6792
Image 6	82.5795	83.3654	83.5581	83.2876	93.0784
Image 7	80.1519	80.7832	80.9438	80.7064	91.7434
Image 8	79.0656	79.8186	80.2571	80.3544	91.5541
Image 9	80.1606	80.9977	81.7340	81.4631	92.1453
Image 10	79.0378	79.9611	80.6182	80.3943	91.5958
Image 11	79.9755	80.6621	81.6421	81.2411	92.1061
Image 12	81.5238	82.4514	83.1347	83.0334	92.8988
Image 13	80.2806	81.2545	81.9351	81.8905	92.3198
Image 14	79.3023	79.9876	80.9226	80.9025	91.8721
Image 15	79.7600	80.7248	81.5586	81.6669	92.1649
Image 16	79.2121	80.1291	81.0836	81.0896	91.9433
Image 17	79.2229	80.3332	81.2824	81.0209	91.9531
Image 18	80.7020	81.7792	81.8114	81.8081	92.3418
Image 19	81.0816	81.8439	82.0954	82.0213	92.4662
Image 20	80.1831	81.0973	81.1744	81.2033	91.9938

**Table 8:** Comparative analysis of accuracy of the projected method with various methods.

Various methods	Accuracy (%)
PSO, GA, and SVM algorithm [18]	89.50
K-NN classification using GA [19]	90.00
Projected GCPSO method	95.81

## Data Availability

The CT images data used to support the findings of this study have been deposited in the LungCT-Diagnosis repository (doi.org/10.7937/K9/TCIA.2015.A6V7JIWX).

## References

[1] Brindha A. A., Indirani S., Srinivasan A. (2016). Lung cancer detection using SVM algorithm and optimization techniques. *Journal of Chemical and Pharmaceutical Sciences*.

[2] Kurkure M., Thakare A. (2016). Introducing automated system for lung cancer detection using Evolutionary Approach. *International Journal of Engineering and Computer Science*.

[3] Rani B., Goel A. K., Kaur R. (2016). A modified approach for lung cancer detection using bacterial forging optimization algorithm. *International Journal of Scientific Research Engineering and Technology*.

[4] Panpaliya N., Tadas N., Bobade S., Aglawe R., Gudadhe A. (2015). A survey on early detection and prediction of lung cancer. *International Journal of Computer Science and Mobile Computing*.

[5] Gupta G. (2011). Algorithm for image processing using improved median filter and comparison of mean, median and improved median filter. *International Journal of Soft Computing and Engineering*.

[6] Tutorial point with digital image processing. http://www.tutorialspoint.com/dip/histogram_equalization.htm.

[7] Venkatalakshmi K., Shalinie S. M. Classification of multispectral images using support vector machines based on PSO and K-means clustering.

[8] Venkatalakshmi K., Shalinie S. M. (2007). Multispectral image classification using modified *k*-means clustering. *International Journal on Neural and Mass-Parallel Computing and Information Systems*.

[9] Venkatalakshmi K., Anisha Praisy P., Maragathavalli R., Mercy Shalinie S. (2007). Multispectral image clustering using enhanced genetic *k*-means algorithm. *Information Technology Journal*.

[10] Dalatu P. I. (2016). Time complexity of *k*-means and *k*-medians clustering algorithms in outliers detection. *Global Journal of Pure and Applied Mathematics*.

[11] Senthilkumar K., Venkatalakshmi K., Karthikeyan K., Kathirkamasundari P. (2015). An efficient method for segmenting digital image using a hybrid model of particle swarm optimization and artificial bee colony algorithm. *International Journal of Applied Engineering Research*.

[12] Venkatalakshmi K., Mercyshalinie S. Classification of multispectral images using support vector machines based on PSO and *k*-means clustering.

[13] Venkatalakshmi K., Anisha Praisy P., Maragathavalli R., Mercyshalinie S. (2008). A customized particle swarm optimization for classification of multispectral imagery based on feature fusion. *International Arab Journal of Information Technology*.

[14] Bansal J. C., Singh P. K. Inertia weight strategies in particle swarm optimization.

[15] Patel P. K., Sharma V., Gupta K. (2013). Guaranteed convergence particle swarm optimization using Personal Best. *International Journal of Computer Applications*.

[16] Wang X., Ge L., Li X. (2012). Evaluation of filters for EnvisatAsar speckle suppression in pasture area. *ISPRS Annals of the Photogrammetry. Remote Sensing and Spatial Information Sciences*.

[17] Nagaveena H., Devaraj D., Prasanna Kumar S. C. (2013). Vessels segmentation in diabetic retinopathy by adaptive median thresholding. *International Journal of Science and Technology*.

[18] Asuntha A., Singh N., Srinivasan A. (2016). PSO, genetic optimization and SVM algorithm used for lung cancer detection. *Journal of Chemical and Pharmaceutical Research*.

[19] Bhuvaneswari P., Brintha Therese A. (2014). Detection of cancer in lung with K-NN classification using genetic algorithm. *International Conference on Nanomaterials and Technologies*.

